# Leptin produced by obesity-altered adipose stem cells promotes metastasis but not tumorigenesis of triple-negative breast cancer in orthotopic xenograft and patient-derived xenograft models

**DOI:** 10.1186/s13058-019-1153-9

**Published:** 2019-05-22

**Authors:** Rachel A. Sabol, Annie C. Bowles, Alex Côté, Rachel Wise, Benjamen O’Donnell, Margarite D. Matossian, Fokhrul M. Hossain, Hope E. Burks, Luis Del Valle, Lucio Miele, Bridgette M. Collins-Burow, Matthew E. Burow, Bruce A. Bunnell

**Affiliations:** 1 Center for Stem Cell Research and Regenerative Medicine, Tulane University, 1430 Tulane Ave, #8699, New Orleans, LA 70112 USA; 2 Department of Medicine, Section of Hematology and Oncology, Tulane University, New Orleans, LA USA; 3 Department of Genetics, Louisiana State University Health Sciences Center (LSUHSC), New Orleans, LA USA; 4 Stanley S. Scott Cancer Center, Louisiana Cancer Research Center (LCRC), LSUSHC, New Orleans, LA USA; 5 Department of Pathology, Louisiana State University Health Sciences Center (LSUHSC), New Orleans, LA USA; 6 Department of Pharmacology, Tulane University, New Orleans, LA USA

**Keywords:** Adipose stem cells, Obesity, Triple-negative breast cancer, Metastasis, Patient-derived xenograft

## Abstract

**Background:**

Breast cancer is the second leading cause of cancer deaths in the USA. Triple-negative breast cancer (TNBC) is a clinically aggressive subtype of breast cancer with high rates of metastasis, tumor recurrence, and resistance to therapeutics. Obesity, defined by a high body mass index (BMI), is an established risk factor for breast cancer. Women with a high BMI have increased incidence and mortality of breast cancer; however, the mechanisms(s) by which obesity promotes tumor progression are not well understood.

**Methods:**

In this study, obesity-altered adipose stem cells (obASCs) were used to evaluate obesity-mediated effects of TNBC. Both in vitro and in vivo analyses of TNBC cell lines were co-cultured with six pooled donors of obASCs (BMI > 30) or ASCs isolated from lean women (lnASCs) (BMI < 25).

**Results:**

We found that obASCs promote a pro-metastatic phenotype by upregulating genes associated with epithelial-to-mesenchymal transition and promoting migration in vitro. We confirmed our findings using a TNBC patient-derived xenograft (PDX) model. PDX tumors grown in the presence of obASCS in SCID/beige mice had increased circulating HLA1^+^ human cells as well as increased numbers of CD44^+^CD24^−^ cancer stem cells in the peripheral blood. Exposure of the TNBC PDX to obASCs also increased the formation of metastases. The knockdown of leptin expression in obASCs suppressed the pro-metastatic effects of obASCs.

**Conclusions:**

Leptin signaling is a potential mechanism through which obASCs promote metastasis of TNBC in both in vitro and in vivo analyses.

**Electronic supplementary material:**

The online version of this article (10.1186/s13058-019-1153-9) contains supplementary material, which is available to authorized users.

## Background

Obesity increases both the incidence and mortality rate of breast cancer in postmenopausal women and is regarded as an important clinical risk factor [1]. Overall, obese patients are diagnosed with larger primary tumors and increased incidence of lymph node metastasis for all subtypes of breast cancers [2]. For women in the USA, breast cancer is both the most frequently diagnosed cancer and the second leading cause of cancer-related. With obesity rates in the USA rising, an increase in the incidence of obesity-associated cancers including breast, endometrial, pancreatic, and colon cancers has also been noted [3]. Therefore, there is a critical need to understand the link(s) between obesity and breast cancer progression.

Triple-negative breast cancers (TNBCs) are a clinically aggressive breast cancer subtype associated with higher mortality. Patients with TNBC are likely to be younger when they develop breast cancer and are more likely to have a recurrence and metastasis within the first 3 years [4, 5]. TNBCs are defined by the lack of targetable receptors (estrogen receptor, progesterone receptor, and EGFR2 amplification). Commonly used small-molecule targeted therapies have been developed for these receptors and are used to treat other cancer subtypes. TNBCs cannot be treated with this approach, resulting in higher rates of recurrence and metastasis, making non-TNBCs more challenging to treat with targeted therapies. Patients with TNBC have worse 5-year survival rates than patients with other BC subtypes [6].

Metastasis accounts for 90% of tumor-related deaths, and an estimated 20–30% of women diagnosed with invasive breast cancer will have recurrence [7]. Metastasis is driven by many different mechanisms. Epithelial-to-mesenchymal transition (EMT) is one proposed mechanism responsible not only for the acquisition of metastasis but can also contribute to resistance to chemotherapy and radiation [8]. During EMT, luminal cells lose epithelial characteristics and gain mesenchymal features, phenotypes that are based on molecular characteristics of the cells as well as cell morphology. This transition facilitates progression to a more invasive and clinically aggressive phenotype.

Obesity is as an independent modifiable risk factor for TNBC that increases the risk of disease progression [9]. The prevalence of obesity has dramatically increased over the past four decades, which has directly correlated to the increased incidence of obesity-associated cancers [10]. Obesity is defined as a body mass index (BMI) greater than 30. Women with a BMI > 30 have an increased incidence and mortality of breast cancer [11]. Specifically, obese women have a 20–40% increased risk of developing breast cancer compared to women with BMI in the healthy weight category [12]. While the link between obesity and breast cancer has been under intense investigation, the cellular mechanisms that are responsible for this association are not fully understood.

Prior studies have determined that obesity biologically alters adipose stem cells (ASCs), an adipose tissue resident stem cell population that is recruited to sites of inflammation including tumors [13–15]. ASCs from obese individuals (obASCs) have a markedly altered biological profile compared to those from lean individuals (lnASCs) and have been shown to produce significantly increased levels of the adipokine leptin [16, 17]. Analysis of cytokine expression demonstrated a pro-inflammatory profile in obASCs compared to lnASCs [13]. Our group has previously reported that ASCs from human lipoaspirate from obese donors (BMI > 30) promote tumor growth and metastasis in ER^+^ breast cancer through, at least in part, a leptin-estrogen pathway [16, 17]. Aromatase, responsible for the biosynthesis of estrogen, is a downstream target of leptin [16, 17]. Therefore, we concluded that increased leptin production by obASCs promotes ER^+^ breast cancer through estrogen-mediated pathways [16–18]. However, this conclusion does not explain the increase of TNBC in obese patients [9, 19], and suggests possible alternative mechanisms through which obASCs promote breast cancer progression. The interaction(s) between obASCs and TNBC has not been fully characterized, but we hypothesize that obASCs will promote TNBC metastasis and provide insight into estrogen-independent pathways through which obesity promotes metastasis.

Patient-derived xenograft (PDX) models have emerged as a novel translational tool for cancer research with the potential to more accurately recapitulate the molecular and behavioral aspects of cancer in the laboratory setting [20, 21]. Traditional in vivo models of breast cancer, specifically the use of immortalized cell lines and orthotopic xenografts, are limited by the lack of tumor heterogeneity. These models use immortalized cell lines that have been used for years, resulting in very high passages in culture and constitute a homogenous, clonal population of breast cancer cells. The long-term culture and passaging of the immortalized cell lines results in irreversible alterations to genetic information and characteristics. Furthermore, these orthotopic xenograft cell line models do not retain tumor tissue architecture or tumor stromal components, which are crucial elements in both tumorigenesis and metastasis. The incorporation of PDX models in our research more directly facilitates the translation of our findings into clinical observations.

Although there is a known association between obesity and TNBC [9, 19], we are among the first to characterize the interaction between obASCs and TNBC. Here, we interrogate this interaction and demonstrate that obesity-altered ASCs promote metastasis of TNBCs through leptin signaling which has implications for discovering novel therapeutic options in a malignancy that has an urgent need for novel targeted therapies.

## Methods

### Adipose human subjects

All protocols were reviewed and approved by the Pennington Biomedical Research Center Institutional Review Board. Subjects provided informed written consent (PBRC #23040). ASCs were isolated from normally discarded adipose tissue from 12 Caucasian females (2 groups, 6 donors/group) undergoing elective liposuction procedures, as previously described [16, 17]. ASCs were isolated from lipoaspirate of subcutaneous adipose tissue isolated from obese women (BMI > 30) or lean women (BMI < 25). Lipoaspirate was washed with phosphate-buffered saline (PBS), incubated at 37 °C in a rocking incubator at 100 rpm for 1 h in 0.1% collagenase type 1 (Sigma, St. Louis, MO, USA), and 1% powdered bovine serum albumin (Sigma) dissolved in 1 ml/g tissue in PBS. Digested tissue was then centrifuged to remove lipids, primary adipocytes, and collagenase solution leaving behind the stromal vascular fraction in the cell pellet. Cells were resuspended in complete culture media (CCM), which consisted of α-minimal essential media (αMEM; Gibco; Grand Island, NY, USA), 10% fetal bovine serum (Atlanta Biologicals, Lawrenceville GA, USA), 100 units per mL penicillin/100μg/mL streptomycin (P/S; Gibco), and 2 mM l-glutamine (Gibco) and plated on T175 culture flasks. Media was replaced every 3 to 4 days until the cells achieved 70% confluence. At 70% confluence, cells were harvested with 0.25% trypsin/1mMEDTA (Gibco) and cryopreserved in liquid nitrogen. The average BMI for the donor groups is as follows: lnASCs (22.7 ± 1.9; *n* = 6) obASCs (32.7 ± 3.7; *n* = 6).

### Cell culture

#### ASCs

Frozen vials of ASCs were thawed and cultured on 150-cm^2^ dishes (Nunc, Rochester, NY, USA) in 20 mL CCM and incubated at 37 °C with 5% humidified CO_2_. After 24 h, plates were washed with PBS and viable cells continued growing with media changes every 3 to 4 days. For all experiments, sub-confluent cells (< 70% confluent) between passages 2 and 6 were used. ASCs were characterized as previously described [17]. Stable transfection of obASCs with a construct targeting leptin and a construct targeting a non-human gene as a negative control was performed as previously described [16].

### Breast cancer cell lines

BT20, MDA-MB-231, MDA-MB-468, MCF7, and HCC1806 cells were purchased from American Type Culture Collection (ATCC; Manassas, VA, USA). BT20, MDA-MB-231, MDA-MB-468, and MCF-7 cells were cultured in αMEM CCM, and HCC1806 were cultured in RPMI (Gibco) with 10% FBS, 1% l-glutamine, and 1% P/S as per ATCC recommendations. Cells were grown at 37 °C in 5% humidified CO_2_, with media changed every 3–4 days, and passaged when cells reached 80–90% confluence.

### Conditioned media proliferation assay

ASCs were plated on a 150-cm^2^ dish and allowed to reach 70% confluence. Then, plates were washed with sterile PBS and medium was replaced with serum-free αMEM for 24 h*. Media* was collected and filtered through a cell strainer (40-μm nylon mesh; Fisher Scientific, Hampton, NH, USA) to remove cellular debris. ASC donors were kept separate and media of six pooled donors was combined for each condition. When culturing TNBC cell lines with ASC conditioned media (ASC CM), TNBC cells were plated at 200 cells per well of a 96-well plate in triplicate in CCM and allowed to adhere overnight. Cells were then washed with PBS and 200 μL lean or obese ASC CM, or serum-free αMEM was added. Proliferation assay was conducted with 10% Alamar blue reagent (Invitrogen, Carlsbad, CA, USA) per manufacturer’s instructions. Proliferation quantification was done by measuring relative fluorescence (excitation 530–560 nm; emission 590 nm).

### Migration assay

CCM or 0.5 × 10^6^ ASCs in CCM were plated in the bottom of a 6-well plate and allowed to adhere overnight. 0.5 × 10^6^ breast cancer cells were seeded in transwells (.4-μm pore; Corning) and allowed to adhere overnight. After 24 h transwells were transferred to wells with CCM or ASCs in CCM and cultured for 3 days. Transwells were then fixed and stained with 3% crystal violet in methanol for 30 min, washed with deionized water, and imaged. Cells were counted with ImageJ.

### Quantitative real time PCR (RT-qPCR)

Six pooled donors of lean or obese ASCs were seeded on top of a transwell migration chamber (4-μm pore) (Corning Inc., Corning, NY, USA). Breast cancer cells were plated in 6-well plates in CCM. Cells were allowed to adhere overnight. Transwell inserts containing ASCs were then transferred to wells with breast cancer cells, or as a control, breast cancer cells were cultured alone for 3 days. After 3 days, breast cancer cells were collected for analysis. RNA was isolated with Qiazol reagent (Qiagen, Valencia, CA, USA) followed by RNeasy columns (Qiagen) and purified by DNase 1 (Qiagen). VILO cDNA synthesis kit (Invitrogen) was used to synthesize cDNA from 1 μg of cellular RNA. RT-qPCR was performed using EXPRESS SYBR Green qPCR SuperMix (Invitrogen). All qPCR data was calculated and reported as the ΔΔCt values that were normalized to the control group for quantitative comparison of mRNA expression levels. Heat map was generated using R coding software gplots library heatmap.2 (open source) with fold change values < 1 as gradient blue and fold change values from 1.5–8 as gradient red [22].

### Orthotopic xenograft model

SCID/beige (CB17.Cg-Prkdc^scid^Lyst^bg-1^/Crl) female mice (4–6-week-old) were obtained from Charles River Laboratory (Wilmington, MA, USA). All protocols involving animals were conducted in compliance with State and Federal law and approved by Tulane University Institutional Animal Care and Use Committee (IACUC). Mice were divided into three groups, with five animals per group: BT20 alone, BT20 with six pooled donors of lnASCs, or BT20 with six pooled donors of obASCs. Cells (1 × 10^6^ per injection) were suspended in 50 μl of PBS and 100 μl phenol-free growth factor reduced Matrigel (BD Biosciences, MA, USA) and injected bilaterally into the mammary fat pads. Animals were anesthetized with isoflurane gas and oxygen delivered by nose cone. Tumor size was measured every 3 to 4 days using digital calipers and calculated as previously described [16]. At necropsy, tissue was collected for further analysis.

### Tumor histology

Harvested tissue was formalin-fixed paraffin embedded (FFPE) and sectioned at a thickness of 5 μm. For hematoxylin and eosin (H & E) staining, slides were deparaffinization and rehydrated and stained with hematoxylin and eosin (Thermo Scientific). For immunohistochemistry, tissue was deparaffinized and rehydrated with Histochoice through descending grades of alcohol to water. 1x citrate buffer pH of 6 (Sigma) was used for heat-mediated antigen retrieval. Tissues were blocked with 1% BSA in TBS-T at room temperature for 30 min in a humidified chamber and stained with primary antibodies against Ki-67 (Cat #: ab15580) (Abcam, Cambridge, UK) diluted 1:200 in 1% BSA in TBS-T or CD31 (Cat #: ab28364) (Abcam) diluted 1:50 1% BSA in TBS-T or HLA (Cat #: ab70328) (Abcam) diluted 1:50 in 1% BSA in TBS-T overnight in a humidified chamber at 4 °C. Sections were washed with TBS and incubated with HRP conjugated secondary for 1 at room temperature in a humidified chamber. ImmPACT DAB reagent (Vector Labs, Burlingame, CA, USA) was used per manufacturer’s instructions to for colorimetric reaction. Slides were washed with PBS and counterstained with hematoxylin or light green. Sections were then dehydrated through ascending grades of alcohol to water and cover slipped using Permount Mounting Medium (Fisher Scientific). Quantification of Ki67 percent positivity was assessed using ImageScope (Aperio, Vista, CA, USA).

Double-label immunofluorescence staining was performed on paraffin-embedded tissue sections according to the standard protocol of LSUHSC Molecular Histopathology and Analytical Microscopy Core. Briefly, paraffin-embedded tissue sections were deparaffinization in xylene, re-hydration through descending grades of alcohol to water, non-enzymatic antigen retrieval in citrate buffer, and then washed by PBS and followed by blocking. First, primary antibody was added to the tissue sections and incubated overnight at room temperature, rinsed in PBS, and a fluorescein-conjugated secondary antibody (1:200 dilution; Invitrogen) was added and incubated for an hour in the dark. After washing with PBS, a second primary antibody was added overnight, followed by rinsing with PBS and incubation with a second rhodamine-tagged secondary antibody (1:200 dilution; Invitrogen) for an hour in the dark. Primary antibodies included mouse monoclonal antibodies against carnitine palmitoyltransferase 1 (CPT1) (Cat #: ab128568) (Abcam), mouse monoclonal against CD44 (Cat #: ab6124) (Abcam), and rabbit polyclonal CD36 (Cat #: ab124515) (Abcam). Sections were then washed in PBS and analyzed using a Confocal Microscope (Olympus FV1000).

### Patient-derived xenograft model

The PDX model used in this study, TU-BcX-2 K1, was derived from the biopsy specimen of an African-American patient that had node negative invasive ductal carcinoma at the time of biopsy. Tumor tissue was obtained through the Louisiana Cancer Research Consortium (LCRC) Biospecimen Core in compliance with NIH regulations and institutional guidelines and approved by the Institutional Review Board at Tulane University and LCRC. All animal procedures were reviewed and approved by Tulane University IACUC. SCID/beige (CB17.Cg-Prkdc^scid^Lyst^bg-1^/Crl) 4–6-week-old female mice were obtained from Charles River Laboratory. Intact tumor pieces were removed and sliced with a scalpel to 3 mm × 3 mm and coated with 100 μL phenol-free growth factor reduced Matrigel (BD Biosciences). In indicated groups, 10^6^ pooled donors (*n* = 6) of lnASCs or obASCs were resuspended in Matrigel and coated the tumor. Tumors were implanted into the mammary fat pads bilaterally under isoflurane and oxygen anesthesia delivered by mask and animals were given 5 mg/kg/day meloxicam for 3 days post-surgery. Tumors were measured by digital caliper every three to 4 days. At endpoint (tumors reach 750–1000 mm^3^), blood, lungs, and tumor were collected for analysis.

### Western blots

Cells were lysed in RIPA buffer (Pierce, Thermo Scientific, Rockford, IL, USA) with phosphatase and protease inhibitors (Pierce). Lysate was sonicated for 10 s followed by 30 s on ice for 3 cycles. Thirty-microgram was fractionated on 4–12% SDS polyacrylamide gels (Invitrogen) and transferred to nitrocellulose membrane (Invitrogen). The blots were blocked in 5% milk in PBS (Sigma) for 1 h at room temperature and probed using primary antibodies incubated overnight at 4 °C, washed with TBS with 0.01% Tween-20 (TBS-T) (Fisher), followed by a secondary antibody conjugated to horseradish peroxidase (HRP), washed with TBS-T three times, and visualized with a chemiluminescent agent (Bio-Rad Laboratories, Hercules, CA, USA) on ChemiDoc MP Imaging System (Bio-Rad). Antibodies used included Phopho-p38 (P-MAPK) (Cat #: 9215S) (Cell Signaling Technology, Danvers, MA, USA), Phospho-AKT (Cat #: 4060) (Cell Signaling Technology), GAPDH (Cat #: 5174), and Leptin (Cat #: PAI-051) (ThermoFisher).

### Flow cytometry

To identify circulating tumor cells, whole blood was collected with 0.5 M EDTA (Gibco). Samples were incubated with 0.008% NH_4_CL for red blood cell lysis and washed with PBS. Cells were then blocked with 1% BSA and 1% CD16/CD32 in PBS and stained with antibodies against HLA1 (Cat #: MA1-80014) (Invitrogen), CD24 (Cat #: 17-0247-41) (EBioscience), CD44 (Cat #: 61-0441-82) (EBioscience), CD326 (Cat #: 46-9326-42) (EBioscience), CD11b (Cat #: 15-0112-81) (EBioscience), CD86 (Cat #: 25-0862-80) (EBioscience), CD206 (Cat #: 12-2061-80) (EBioscience). Samples were analyzed with a Gallios Flow Cytometer (Beckman Coulter, Brea, CA, USA) with Kaluza software (Beckman Coulter). A minimum of 10,000 events were captured and analyzed. Flow gating strategy is represented in Additional file 7: Figure S7.

### Statistical analysis

All data are represented as mean ± SEM. Analysis of variance (ANOVA) was used for comparison of three groups with Tukey post-hoc analysis. Statistical significance was set at *p* < 0.05. Analysis was performed using GraphPad Prism software.

## Results

### ASC secretome enhances proliferation and migration of TNBC cell lines

**Fig. 1:**
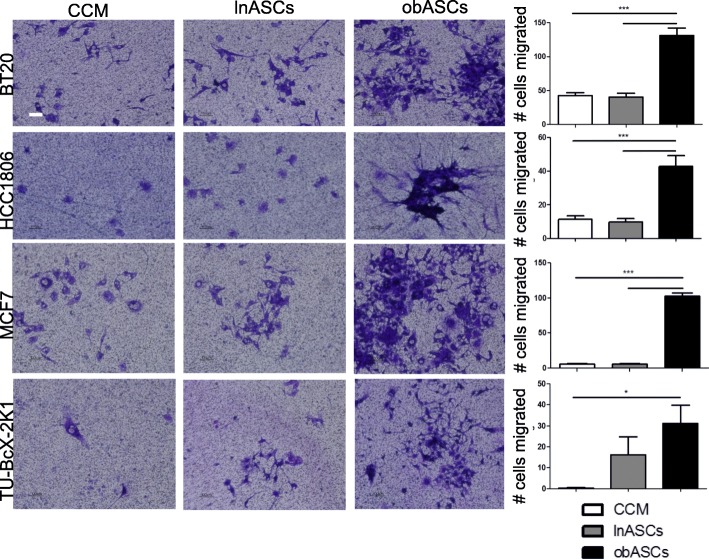
Secreted factors from adipose stem cells promotes migration of breast cancer. obASCs promoted increased migration of BT20, MCC1806, and MCF7, patient-derived xenograft TU-BcX-2 K1-derived cells through a 0.4-um membrane. All images were acquired at the same magnification. Scale bar represents 100 μm. Values reported are the mean of three independent experiments each performed in triplicate. Bars, ± SEM. *p < 0.05, **p < 0.01, ***p < 0.001

Adipose stem cells secrete numerous immunomodulatory and growth factors [13]. We have previously reported that obASC conditioned media enhanced the proliferation of the ER^+^ breast cancer cell line MCF7 [17]. To evaluate the effect of the ASC secretome on TNBC proliferation, TNBC cell lines (BT20, HCC1806, MDA-MB-468, MDA-MB-231) were cultured in ASC conditioned media (CM) from both lnASCs and obASCs and compared to TNBC proliferation rates in serum-free (SF) medium as a control. BT20 cultured with obASC CM produced 4683 ± 579 relative fluorescent units (RFU) with Alamar blue on day four compared to 3237 ± 378 (mean ± SEM) with lnASC CM and 739 ± 41 RFU with SF medium (Additional file 1: Figure S1). ASC conditioned media similarly stimulated proliferation of TNBC cell lines: HCC1806, MDA-MB-231, and MDA-MB-468 (Additional file 1: Figure S1). Additionally, a migration assay was performed in a transwell system with stem cells plated in the well and cancer cells seeded in the transwell insert. The non-invasive ER^+^ breast cancer (MCF7) and Basal-A TNBC (BT20 and HCC1806) cell lines had significantly increased migration to the obASCs (BT20: 131.3 ± 10.9 migrated cells) compared to lnASCs (BT20: 40.9 ± 5.4) or CCM (BT20: 42.5 ± 4.3) (Fig. 1). TNBC PDX-derived cells from TU-BcX-2 K1 migration were also evaluated. PDX cell migration was significantly increased in obASCs (31.2 cells ± 8.6; mean ± SEM) compared to CCM (0.3 cells ± 0.2) and demonstrated a trend of increased migration over lnASCs (16.2 cells ± 8.6) (Fig. 1). These data indicate that the obASC secreted factors promote a migratory breast cancer phenotype.

### obASCs alter the gene expression profile of luminal/basal-a breast cancer cell lines

**Fig. 2:**
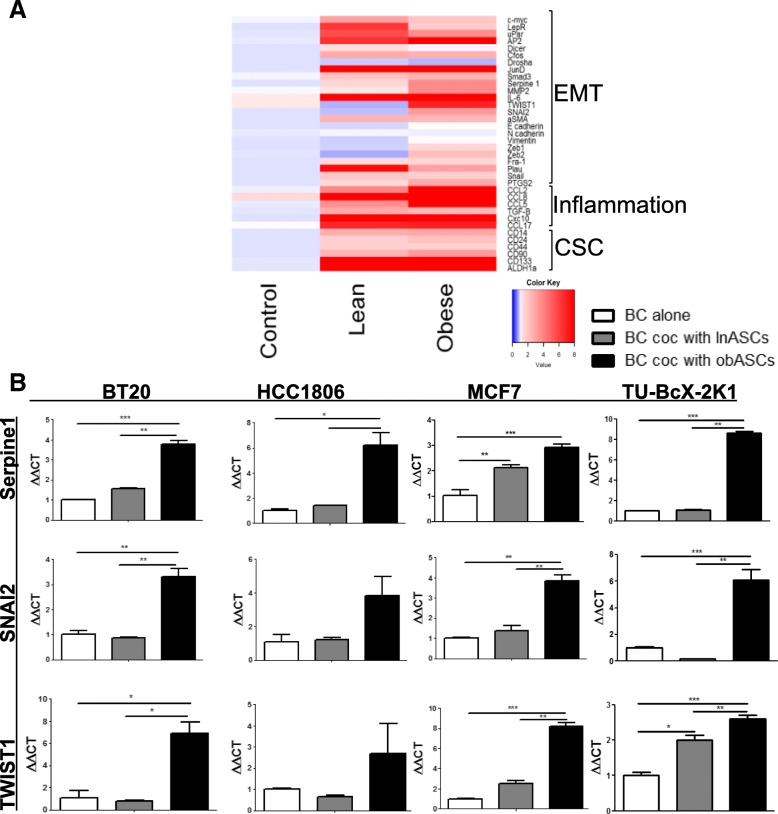
Transwell co-culture of breast cancer cells with obASCs increases expression of metastatic genes. **a** TNBC cell line BT20 gene expression was screened for changes in epithelial-to-mesenchymal transition (EMT), inflammatory, and cancer stem cell (CSC) genes after 96-h of transwell co-culture (shown in heat map). **b** Serpine1, TWIST1, and SNAI2 were evaluated across four cell lines: BT20, HCC1806, MCF7, and TU-BcX-2 K1 PDX-derived cells. Values reported are the mean of three independent experiments each performed in triplicate. Bars, ± SEM. *p < 0.05, **p < 0.01, ***p < 0.001

TNBC cell line BT20 was co-cultured for 96 h with lean or obesity-altered ASCs using transwells. Changes in the expression of mRNA of genes associated with breast cancer progression were quantified using RT-qPCR. The genes represent pathways such as epithelial-to-mesenchymal transition (EMT), inflammatory signaling, and cancer stem cells (CSCs). The genes analyzed were *MYC*, *OBR*, *UPAR*, *AP2*, *DICER*, *CFOS*, *DROSHA*, *JUND*, *SMAD3*, *SERPINE-1*, *MMP2*, *IL-6*, *TWIST1*, *SNAI2*, *ACTA2*, *CDH1*, *CDH2*, *VIM*, *ZEB1*, *ZEB2*, *FRA1*, *PLAU*, *SNAIL*, *PTGS2*, *CCL2*, *CCL8*, *CCL5*, *TGFB*, *CXC10*, *CCL17*, *CD14*, *CD24*, *CD44*, *CD90*, *CD133*, and *ALDH1A*. Transwell co-culture with obASCs resulted in enrichment of mRNA expression of various metastasis-associated genes (Fig. 2a). However, obASCs also downregulated the expression of the leptin receptor in BCCs. It is likely that this is due to the negative feedback of high levels of leptin produced by obASCs (Fig. 2a). From the gene panel evaluated in BT20, three migration/metastasis-related genes that had previously been described to be upregulated by obASCs in ER^+^BC were evaluated across three immortalized cell lines and PDX-derived cells: SERPINE1, SNAI2, and TWIST1. obASCs upregulated the expression of Serpine1 (3.79 fold ± 0.18), TWIST1 (6.86 fold ± 1.08), and SNAI2 (3.30 fold ± 0.34) in BT20 over control while co-culture with lnASCs resulted 1.56 fold ± 0.02 increase in Serpine1, 0.79 fold ± 0.02 decrease in TWIST1, and 0.86 fold ± 0.04 change in SNAI2. Exposure of PDX-derived cancers cells to obASCs resulted in similar expression changes of Serpine1, TWIST1, and SNAI2 (8.61 fold ± 0.19, 2.60 fold ± 1.40, 6.11 fold ± 1.15, respectively). Obesity-altered ASCs similarly increased expression of these genes in MCF7 and HCC1806 while ASCs from lean adipose tissue did not promote the expression of these metastasis-related genes (Fig. 2b).

### Obesity-altered ASCs do not enhance tumor growth but promote metastasis of TNBC orthotopic xenograft

**Fig. 3:**
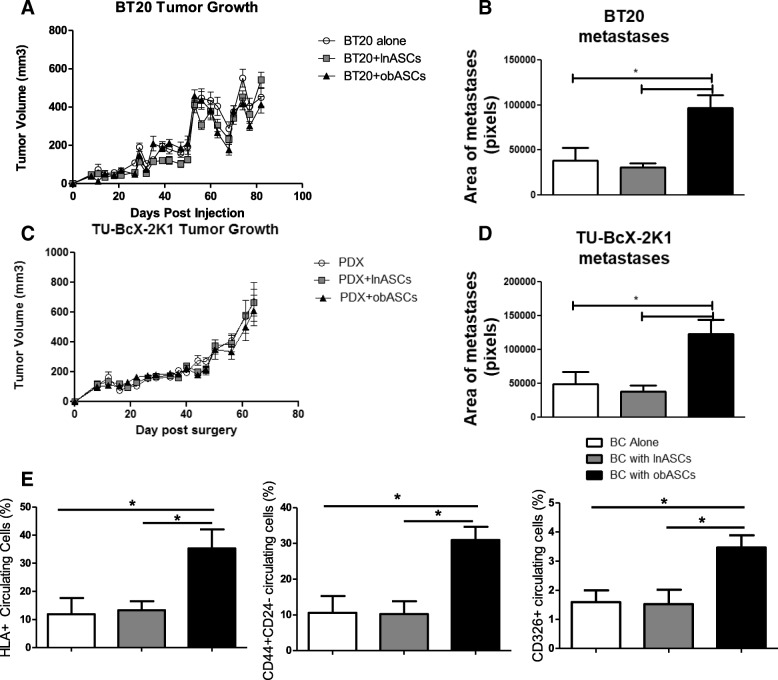
Adipose stem cells do not affect tumor growth of TNBC xenogafts, but obASCs increase metastases. **a** Average tumor volume of TNBC cell line BT20 xenograft was consistent for all groups across the time course of the experiment. **b** Evaluation of metastasis revealed increased area of lung metastases from BT20 tumors grown with obASCs. **c** Average tumor volume of TNBC PDX TU-BcX-2 K1 was consistent for all groups across the time course of the experiment. **d** Evaluation of metastasis revealed increased area of metastases in lungs of mice with PDX tumors grown with obASCs in comparison to lnASCs or PDX only. **e** Flow cytometric analysis of the blood demonstrates increased circulating HLA1^+^ “human cells” in the blood of the obASC group. There is an increase in cancer stem cell marker CD44^+^CD24^−^ in the CTCs from the obASC group. There is an increase in circulating CD326^+^ (epithelial cell adhesion molecule) cells in the obASC group. Caliper measurements were taken every 3 to 4 days until tumor volume reached 750–1000 mm^3^. Values reported are the mean (*n* = 5 mice/group). Bars, ± SEM. *p < 0.05, **p < 0.01, ***p < 0.001

The TNBC cell line (BT20) was used in an orthotopic xenograft model in SCID/Beige immunocompromised mice. BT20 tumors grown in the absence of ASCs were compared to tumors grown with pooled donors of lnASCs or obASCs (*n* = 6). No significant differences in tumor growth were observed over time across all of the groups (Fig. 3a). Immunohistologic analysis of tumors at endpoint revealed no differences in proliferation of tumor cells as quantified by Ki-67^+^ cells (Additional file 2: Figure S2A). Additionally immunostaining for CD31^+^ as an indicator of mature endothelial vasculature revealed no differences in angiogenesis at the termination of the study across tumor groups (Additional file 2: Figure S2A). IHC of lung sections with HLA1 to delineate human metastases demonstrated a significant increase in metastases in mice where tumors were grown with obASCs compared to control and lnASC groups (Fig. 3b, Additional file 2: Figure S2B). Additionally, there was no significant increased metastasis in tumors grown with lnASCs compared to control (Fig. 3b). Together, these data from the orthotopic xenograft experiment show that obASCs promote metastasis of a basal-A TNBC cell line, but do not affect tumor growth, proliferation, or angiogenesis**.**

### TNBC patient-derived xenograft tumor growth is unaffected by obASCs; however, obASCs promote increased circulating tumor cells and metastasis of TNBC PDX

To confirm our findings in a pre-clinical translational model, the PDX model, TU-BcX-2 K1, which was established at Tulane University and represents an invasive ductal carcinoma was used (Additional file 3: Figure S3A). TU-BcX-2 K1 intact PDX tumor pieces were implanted bilaterally into the mammary fat pads of SCID/Beige mice coated in growth factor reduced Matrigel alone or with pooled donors (*n* = 6) of lnASCs or obASCs resuspended in growth factor reduced Matrigel. Evaluation of tumor volume over time revealed the ASCs did not have any effect on the growth of TU-BcX-2 K1 (Fig. 3c). Histological analysis of tumors revealed no change in proliferation as measured by Ki67^+^ cells or angiogenesis as measured by CD31^+^ vessels (Additional file 3: Figure S3B). A significant increase in the area of metastases was observed in the lungs of the mice with TU-BcX-2 K1 TNBC PDX grown with obASCs compared to lnASCs or control groups. Additionally, there was no difference in metastasis in PDX tumors grown with lnASCs compared to control PDX tumors (Fig. 3d, Additional file 4: Figure S4A). Further, flow cytometric analysis of blood for circulating tumor cells (CTCs) revealed a significant increase in HLA^+^ “human” circulating cells in mice with PDX tumors coated with obASCs (Fig. 3e). The frequency of breast cancer CTCs as indicated by CD44^+^CD24^−^ was also significantly increased from PDX tumors with obASCs (Fig. 3e). Due to the increased levels of CTCs (CD44^+^CD24^−^) in the blood of the animals in the obASC-PDX group (Fig. 3e), the expression of CD44 in tumor sections was analyzed using immunohistochemistry. CD44-positive cells also co-expressed fatty acid transporter marker CD36 as well as CPT1, the rate-limiting enzyme of fatty acid oxidation (FAO) pathways (Additional file 3: Figure S3C, D). We also evaluated the frequency of myeloid cells in circulation and found that PDX tumors with obASCs stimulated the myeloid population in these mice, and there was an increase in both M1 (CD11b^+^CD86^+^) macrophages and M2 (CD11b^+^CD206^+^) macrophages in circulation (Additional file 4: Figure S4B). Additionally, we evaluated the expression of the epithelial cell adhesion molecule (CD326) because this protein is downregulated during EMT. Analysis of the PDX tumors from the obASC group demonstrated depletion of CD326 (Additional file 4: Figure S4B), but a significant increase in CD326^+^ CTCs in the blood (Fig. 3e). Taken together, these data demonstrate that obASCs are promoting EMT, through downregulation of CD326 in tumors, increasing HLA^+^ CTCs enriched for the cancer stem cell maker CD44^+^CD24^−^, and have increased lung metastases.

### Leptin knockdown via shRNA abrogates obASC promotion of metastatic potential of breast cancer

**Fig. 4:**
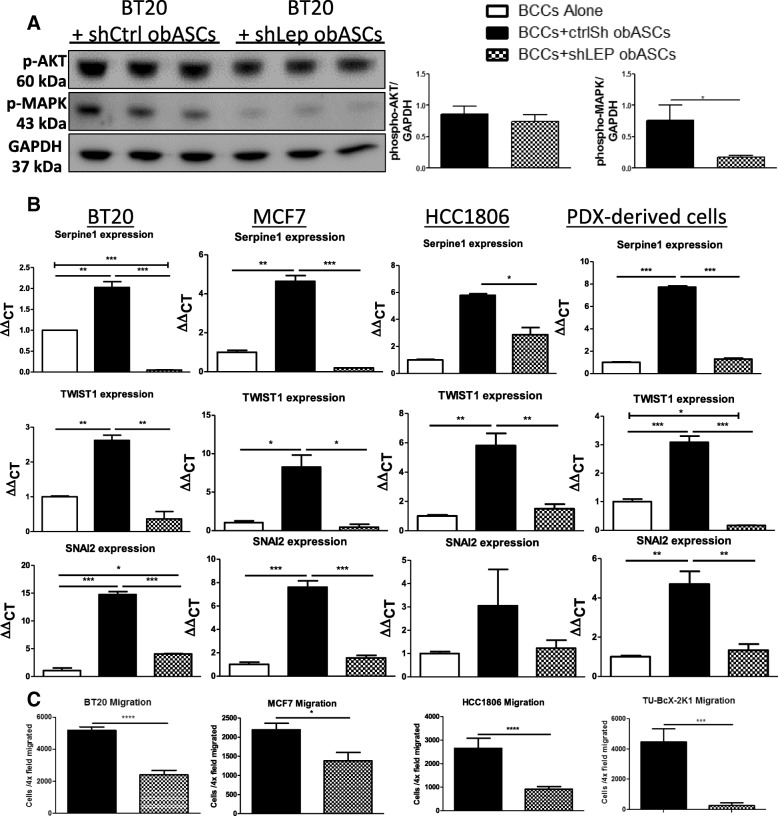
Knockdown of leptin expression in obASCs decreases promotion of metastatic phenotype. **a** Western blots of signaling pathways activated in BCCS after Transwell co-culture with control shRNA obASCs compared to leptin shRNA obASCs. **b** RT-qPCR after transwell co-culture with obASC or obASC with leptin shRNA demonstrates decreased mRNA levels of Serpine1, TWIST1, and SNAI2. **c** Leptin shRNA obASCs decreased migration of BT20, HCC1806, and MCF7 breast cancer cell lines through a 0.4-um transwell membrane. Values reported are the mean of three independent experiments each performed in triplicate. Bars, ± SEM. *p < 0.05, **p < 0.01, ***p < 0.001

To evaluate what factor from obASCs might promote this metastatic phenotype of TNBCs, we evaluated leptin, which has been previously shown to be produced at high levels from obASCs compared to lnASCs [23]. Additionally, it has been previously shown that leptin from obASCs promoted both the growth and metastasis of ER^+^BC through upregulation of aromatase and ERα [16, 17]. We hypothesized that leptin could be mediating the metastatic phenotype observed in these TNBC cell lines and PDX model. Consistent with previous reports, we found that obASCs produce high levels of leptin compared to lnASCs (Additional file 5: Figure S5A). An shRNA strategy was used to knockdown leptin expression in obASCs (shLep obASCs), and a non-human gene as a negative control shRNA construct for control obASCs (shCtrl obASCs), as previously described [16]. RT-qPCR was used to demonstrate the reduced leptin expression as a result of stable transfection of obASCs with leptin shRNA and confirmed via western blot (Additional file 5: Figure S5B). Activation of signaling pathways known to be activated by leptin were evaluated in BCCs co-cultured with shCtrl obASCs or shLep obASCs. We found a significant increase in phosphorylated MAPK in BCCs co-cultured with shCtrl obASCs compared to shLep obASCs. No significant difference in phospho-AKT was found (Fig. 4a). Evaluation of gene expression changes in breast cancer cells after transwell co-culture with shLep obASCs did not upregulate EMT genes compared to shCtrl obASCs. ObASCs with control shRNA increased Serpine1 expression in BT20 (2.02 ± 0.14), MCF7 (4.63 ± 0.30), HCC1806 (5.78 ± 0.12), and 2 K1 PDX-derived cells (7.72 ± 0.11). The gene expression fold change in these cells after transwell co-culture with obASCs with leptin shRNA was 0.04 fold ± 0.0043 (BT20), 0.19 ± 0.0013 (MCF7), 2.87 ± 0.52 (HCC1806), and 1.30 ± 0.08 (2 K1 PDX). Other metastasis-related genes demonstrated similar trends, including TWIST1, SNAI2, CCL5, CD90, PTGS2, and IL-6 (Fig. 4b, Additional file 6: Figure S6). Additionally, shCtrl obASCs upregulated CCL5 in these cell lines compared to control BCCs and cells co-cultured with shLep obASCs. These data are consistent with previous reports demonstrating that adipose tissue promotes invasiveness of TNBC through upregulation of CCL5 [24], and here, we implicate leptin as a signaling mediator of this metastatic phenotype. Finally, migration assays were performed in transwells in which TNBC lines were co-cultured with leptin shRNA knockdown ASCs. Quantification of breast cancer cell migration to leptin shRNA obASCs compared to control shRNA obASCs demonstrated that breast cancer had decreased migratory ability when leptin was knocked down in obASCs (Fig. 4c).

**Fig. 5:**
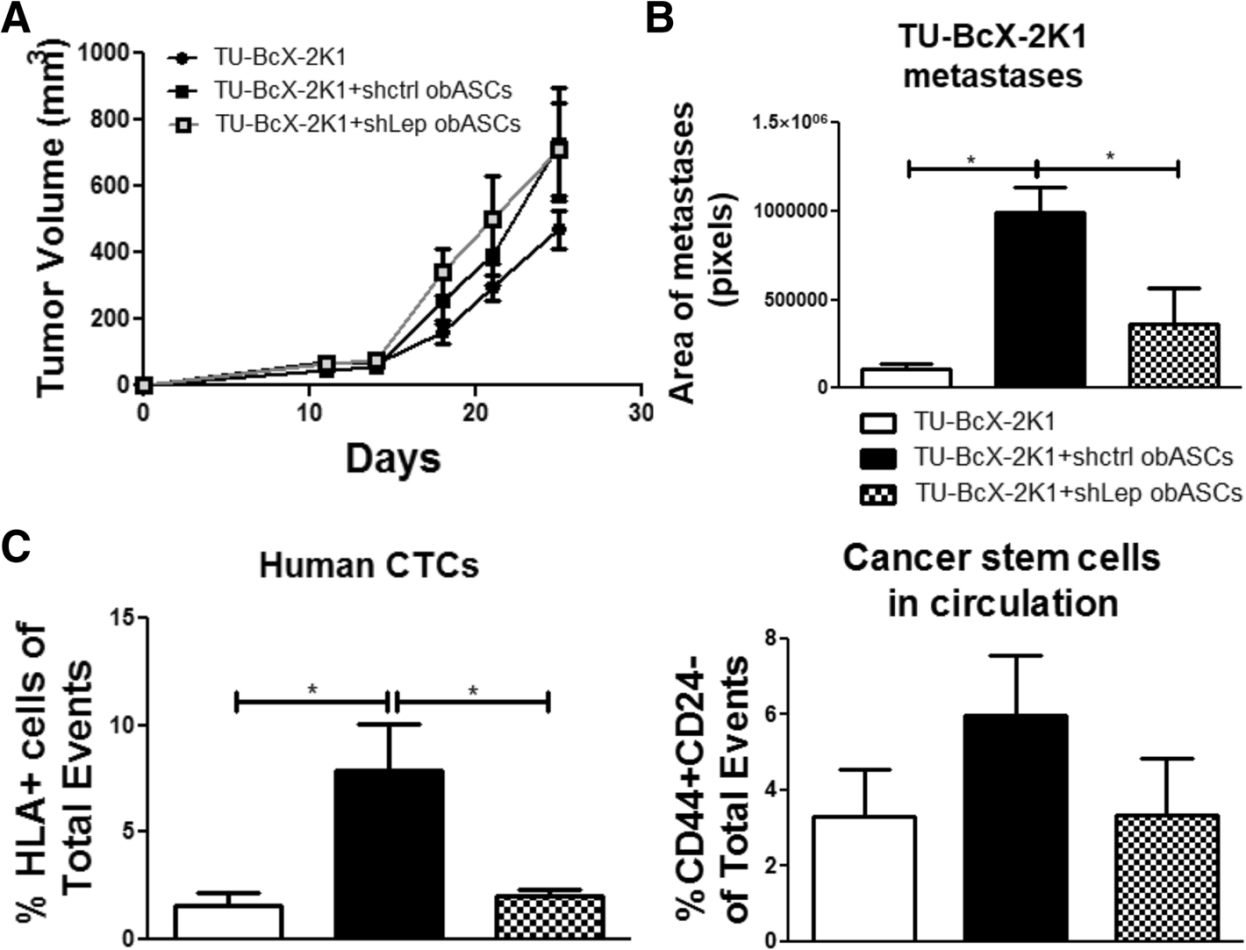
Leptin knockdown abrogates pro-metastatic effect of obASCs in TNBC PDX model. **a** Evaluation of tumor growth over time after implantation demonstrates that there is no difference in tumor growth of TU-BcX-2 K1 grown alone or in the presence of shCtrl obASCs or shLep obASCs. **b** Evaluation of metastases demonstrates that shCtrl obASCs promote metastasis of TNBC PDX model compared to both control and shLep tumors. **c** Evaluation of circulating tumor cells reveals that there is an increased percentage of HLA^+^ circulating tumor cells when tumors are grown with shCtrl obASCs compared to control and shLep obASC groups. Additional evaluation of cancer stem cell markers on circulating tumor cells reveals a trend of an increased percentage of circulating cancer stem-like cells in the shCtrl obASCs group of TNBC PDX compared to control and shLep obASCs. Mean values are represented (*n* = 5 mice/group) Bars, ± SEM. *p < 0.05

Our group implanted TU-BcX-2 K1 TNBC PDX tumors with control shRNA obASCs and shLeptin obASCs to evaluate if leptin from obASCs promoted TNBC metastasis in vivo*.* Evaluation of tumor growth revealed that obASCs and shLep obASCs did not affect tumor growth compared to PDX alone (Fig. 5a). When tumor volume reached 750–1000 mm^3^ animals were sacrificed. Circulating tumor cells were evaluated using flow cytometry and metastases were quantified. We stained lung sections with HLA1 to delineate human cells in the lungs and subsequently quantified metastases. We compared TU-BcX-2 K1 tumors alone, with shCtrl obASCs or with shLep obASCs, and found that shCtrl obASCs had significantly increased area of metastases compared to control tumors or tumors grown with shLep obASCs (Fig. 5b). Finally, we evaluated circulating tumor cells and found that obASCs promoted an increased percentage of HLA1^+^ “human” circulating tumor cells compared to mice with control tumors and tumors with shLep obASCs. Additionally, CTC evaluation of mice with PDX tumors with obASCs demonstrated a trend of an increased percentage of human cells enriched for the cancer stem cell marker CD44^+^CD24^−^ compared to mice with control tumors and tumors with shLeptin obASCs (Fig. 5c). To conclude, this study demonstrates that leptin from obASCs promotes increased human circulating tumor cells and increased metastases in a TNBC PDX model.

## Discussion

Studies focused on TNBC because it is very aggressive, is highly metastatic, and is associated with obesity. The incidence of TNBC in obese women is higher than that in non-obese women [19]. Additionally, obese TNBC patients are reported to have larger tumor size, grade, and stage [19]. TNBC lacks targetable receptors for treatment with targeted therapies, and therefore, the discovery of novel therapies for TNBC is essential. TNBC can be further categorized into luminal and basal-like subtypes, with basal being more clinically aggressive and conferring a worse prognosis. Luminal TNBC is characterized by the expression of cytokeratins 7 and 8 and increases in many hormonally regulated pathways. Basal TNBC is characterized by expression of cytokeratins 5 and 6, as well as high epidermal growth factor receptor expression [25, 26]. Basal subtypes are subclassified as basal-A and basal-B, with basal-B being more invasive and mesenchymal-like [27]. In this study, we used less aggressive, basal-A TNBC-established cell lines and a luminal ER^+^ cell line MCF7 for comparison and observed that obesity-altered ASCs promoted a more aggressive metastatic phenotype in these cells through leptin-mediated pathways.

Previous studies demonstrate that ASCs resident in adipose tissue are recruited to the tumor and promote tumor progression through proteases, pro-angiogenic factors, and adipokine secretions [15–17, 28, 29]. In this study, the impact of obesity-altered ASCs on TNBC was investigated. Here, we found that CM from lean or obesity-altered ASCs promoted BC proliferation, which suggests that the factors secreted by ASCs signal with the TNBC cells to promote proliferation in vitro*.* However, in vivo tumorigenesis experiments with a basal-A TNBC cell line and TU-BcX-2 K1 TNBC PDX indicated that the ASCs had no effect on tumor growth. This outcome is different from what has previously been reported on tumor growth of ER^+^ breast cancer and obASCs, where obASCs promoted tumor growth of ER^+^ breast cancer xenografts. We posit that this observation was the result, in part, of the fact that TNBC at baseline is more proliferative and aggressive than ER^+^ breast cancer and the non-estrogen pathways that promote tumor growth in ER^+^ breast cancer are already activated in TNBC.

While obASCs failed to promote tumor growth of TNBC xenograft and PDX tumor growth, the same cells promoted metastasis of both in vivo. Circulating tumor cell (CTC) analysis in our PDX model revealed a significant increase in human circulating cells (HLA1^+^) enriched for breast cancer stem cell marker (CD44^+^CD24^−^). This increase in circulating human CSCs correlated to enhanced metastases seen when the PDX were co-implanted with obASCs. Evidence supports the role of cancer stem-like cells in tumor metastasis [30–33]. We evaluated PDX tumors for CSC markers through IHC. We found fatty acid transporter, CD36, and the rate-limiting enzyme in fatty acid oxidation, CPT1, expression within CD44^+^ cells, which have been identified as subpopulations of metastasis-initiating cells. Recent studies also suggest the role of extracellular lipid uptake and increased FAO in CSCs [34–36]. Altogether, these data support the finding that obASC-PDX tumor-bearing mice had increased metastasis with elevated circulating CSCs. This is significant because metastasis accounts for 90% of tumor-related deaths in breast cancer patients [7]. The promotion of EMT and a metastatic phenotype in TNBC by obASCs could explain, at a cellular level, the worse prognosis and outcomes for obese patients with TNBC that is seen on an epidemiologic level. Evaluation of potential mechanisms that obASCs employ to promote a metastatic phenotype was performed based on expression changes of EMT, CSC- and inflammation-associated genes and migration assays. We found that obesity-altered ASCs but not their lean counterpart promoted EMT and a metastatic phenotype.

ASCs are known to be a source of leptin, a growth factor, in adipose tissue. It has previously been shown that obASC produce much higher levels of leptin than lnASCs [16, 17]. Previous reports have demonstrated leptin’s ability to promote many cancer signaling pathways including phosphatidylinositol 3 – kinase protein kinase B (PI3K/AKT), mitogen-activated protein kinase (MAPK), and Janus Kinase 2-Signal transducer and activator of transcription 3 (JAK2/STAT3) pathways that promote tumorigenesis and metastasis [16, 37–41]. While leptin treatment has been shown to upregulate Serpine1, TWIST1, and SNAI2, the direct signaling mechanism(s) connecting leptin signaling and upregulation of these factors have not been delineated. Singh et. al. have previously shown that leptin upregulates expression of Serpine1 in vascular epithelial cells [42]. Serpine1 is a serine protease inhibitor shown to play a role in cancer metastasis through binding of vitronectin leading to detachment of cancer cells from the extracellular matrix [43]. Here, we show leptin produced by obASCs increases expression of Serpine1 in TNBC promoting a metastatic breast cancer phenotype. Additionally, obASCs promote increased expression of IL-6. IL-6 is a pro-inflammatory cytokine that is associated with tumor progression [44, 45]. SNAI2 (snail family zinc finger 2) expression is a gene implicated in EMT progression [46]. Leptin treatment of breast cancer cells in vitro has been shown to increase SNAI2 expression [37]. Elevated levels of Serpine1, IL-6, and SNAI2 correlate with the pro-metastatic phenotype of breast cancer cells induced by obASCs in vitro and the increase in metastatic lesions in the lungs of mice with TNBC tumors grown with obASCs.

In this study, obASC stably transfected with a leptin shRNA construct was used to interrogate the mechanism by which obASC-derived leptin alters TNBC biology [16]. While we previously reported that obASCs co-cultured with BC cells promotes a mesenchymal and metastatic cell phenotype based on upregulation of select EMT genes, the data in this manuscript indicate that stable knockdown of leptin through shRNA abrogated these effects. These data suggest that leptin enhances EMT in TNBC through upregulation of TWIST1, SERPINE1, SNAI2, IL-6, PTGS2, CCL5, and CD90. Beyond gene expression alterations, it was determined that knockdown of leptin in obASCs in a transwell migration assay decreased the migration of BC that was stimulated by obASCs. These data suggest that the high levels of leptin produced by obesity-altered ASCs promote metastasis of TNBC through the upregulation of the expression of multiple factors that promote cancer cell migration and metastasis.

## Conclusions

Obesity alters ASCs to acquire tumorigenic characteristics, which are then recruited to the tumor microenvironment and secrete increased levels of leptin, resulting in enhanced metastasis of TNBC through leptin-mediated pathways. Here, it is shown that leptin from obASCs increases expression of Serpine1, SNAI2, IL6, TWIST1, and PTGS2, which are some key players in the EMT and CSC programs in cells in MCF7, BT20, HCC1806, and TU-BcX-2 K1 TNBC PDX-derived cell lines. The results of these studies provide evidence on a cellular level of obesity-mediated promotion of TNBC metastasis via increased levels of leptin produced by obesity-altered ASCs. These data suggest that leptin may be a novel prognostic marker for the worse prognoses seen in clinic for obese patients with TNBC. Future studies that interrogate leptin and its downstream mediators as potential targets for precision medicine in obese TNBC patients are necessary.

## Additional files

Additional file 1: **Figure S1.** Secreted factors from adipose stem cells promotes proliferation and migration of breast cancer. Conditioned media collected from ASCs after 24 h promotes proliferation of MDA-MB-231, MDA-MB-468, BT20, and HCC1806 TNBC cell lines and TU-BcX-2 K1-derived cells. Values reported are the mean of three independent experiments each performed in triplicate. Bars, ± SEM. *p < 0.05, **p < 0.01, ***p < 0.001. *−obASC vs. control, #−lnASCs vs control (PDF 112 kb)

Additional file 2: **Figure S2.** TNBC xenograft growth is unaffected by ASCs. (A) Histologic analysis of tumors at end point revealed that stem cells had no effect on proliferation (ki67+ cells) or angiogenesis (CD31+ blood vessels). All images in panel were acquired at the same magnification. Scale bar represents 100 μm. (B) Representative images of metastases are shown. Image of lung section with annotation metastases are shown. Black arrows point to annotations on lung sections. All images in this panel were acquired at the same magnification. Scale bar represents 100 μm. Values reported are the mean (*n* = 5 mice per group). Bars, ± SEM. *p < 0.05, **p < 0.01, ***p < 0.001. (PDF 671 kb)

Additional file 3: **Figure S3.** Patient-derived xenograft. (A) Primary human TNBC was surgically removed and implanted with Matrigel into SCID/Beige female mice (*n* = 5 per group). SCID/Beige mice lack B cells, T cells, and functional natural killer cells. At passage seven, tumors pieces were implanted with lnASCs, obASCs, or PDX alone. (B) Analysis of immunostaining of tumors at end point revealed that ASCs had no effect on proliferation (ki67^+^ cells) or angiogenesis (CD31^+^ blood vessels). All images in panel acquired at the same magnification. Scale bar represents 200 μm. (C) Histologic analysis CD44^+^ is shown. Scale bar in upper row represents 60 μm and 20 μm in the lower panel. (D) Histologic analysis CD44^+^, CD36^+^, and CPT1 is shown. All images were acquired at the same magnification. Scale bar represents 10 μm. Values reported are the mean (*n* = 5 mice/group). Bars, ± SEM. *p < 0.05, **p < 0.01, ***p < 0.001. (PDF 10977 kb)

Additional file 4: **Figure S4.** Flow cytometric analysis of PDX tumor shows obASCs promote EMT and increase classically activated macrophages and alternatively activated macrophages. (A) Representative images of metastases are shown all and were acquired at the same magnification (scale bar = 100 μm). Image of lung section with annotation metastases are shown. Black arrows point to annotations on lung sections. (B) PDX tumors were digested using 0.01% collagenase to a single-cell suspension, and cells were analyzed using flow cytometry. Tumors had no significant change in cancer stem cell enrichment marker CD44^+^CD24^−^, but tumors from the obASC group showed decreased expression of CD326 (epithelial cell adhesion molecule). Analysis of myeloid cells in circulation revealed that obASCs increase the circulating myeloid cells (CD11b^+^). obASCs significantly increase classically activated macrophages (CD11b^+^CD86^+^) and alternatively activated macrophages (CD11b^+^CD206^+^) cells. Mean values are represented (*n* = 5 mice/group) Bars, ± SEM. *p < 0.05. (PDF 1452 kb)

Additional file 5: **Figure S5.** shRNA knockdown of leptin in obASCs. (A) Representative images of metastases are shown and were acquired at identical magnification (scale bar = 100 μm). (B) Leptin expression in control shRNA obASCs versus leptin shRNA obASCs was compared to evaluate knockdown efficiency. Leptin knockdown was confirmed via Western blot. Values reported are the mean of three independent experiments each performed in triplicate. Bars, ± SEM. *p < 0.05, **p < 0.01. (PDF 278 kb)

Additional file 6: **Figure S6.** shRNA knockdown of leptin in obASCs decreases pro-metastatic gene expression in breast cancer cells. RT-qPCR of breast cancer cells after 3 days of transwell co-culture with control shRNA obASCs or leptin shRNA obASCs shows an increase in expression of CCL5, CD90, PTGS2, and IL-6 after co-culture with obASCs that is abrogated by leptin shRNA in the obASCs. Values reported are the mean of three independent experiments each performed in triplicate. Bars, ± SEM. *p < 0.05, **p < 0.01, ***p < 0.001. (PDF 182 kb)

Additional file 7: **Figure S7.** Flow cytometry gating strategy. Representative dot plots and histograms generated from Beckman Coulter Galios Flow Cytometer are shown here to represent the gating strategies for flow cytometry analysis included in this manuscript. (PDF 361 kb)

## Abbreviations

ASCs: Adipose stem cells
BMI: Body mass index
CCM: Complete culture media
CM: Conditioned media
CPT1: Carnitine palmitoyltransferase 1
CSCs: Cancer stem cells
CTCs: Circulating tumor cells
EMT: Epithelial-to-mesenchymal transition
FAO: Fatty acid oxidation
FFPE: Formalin-fixed paraffin embedded
H&E: Hematoxylin and eosin
JAK2/STAT3: Janus kinase2-signal transducer and activator of transcription 3
LCRC: Louisiana Cancer Research Consortium
lnASCs: Lean adipose stem cells
MAPK: Mitogen-activated protein kinase
obASCs: obesity-altered adipose stem cells
PBS: Phosphate-buffered saline
PDX: Patient-derived xenograft
PI3K/AKT: Phosphatidylinositol 3 – kinase protein kinase B
RFU: Relative fluorescent units
SF: Serum free
TNBC: Triple-negative breast cancer
αMEM: Alpha minimal essential media
