# Supplementary figures and images for: Leptin produced by obesity-altered adipose stem cells promotes metastasis but not tumorigenesis of triple-negative breast cancer in orthotopic xenograft and patient-derived xenograft models
Source: Breast Cancer Res. 2019 May 22;21:67. doi: 10.1186/s13058-019-1153-9 (PMC6530039; doi:10.1186/s13058-019-1153-9)

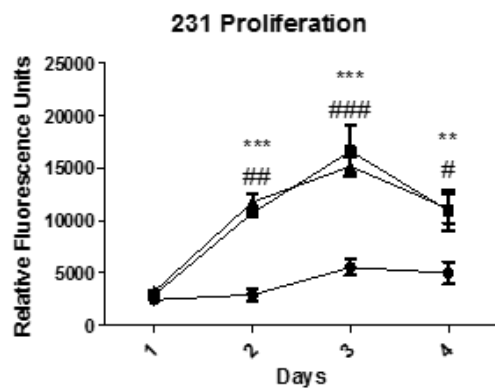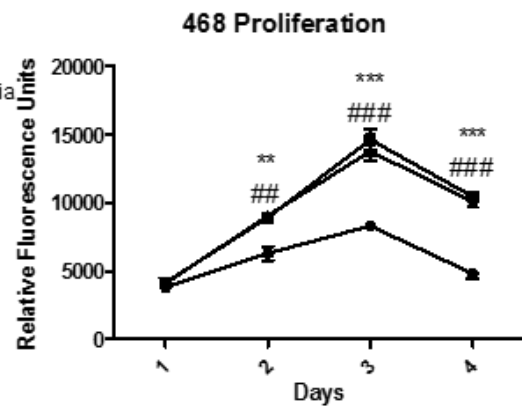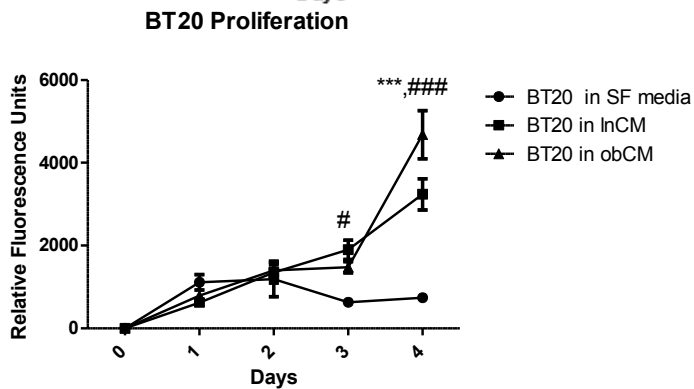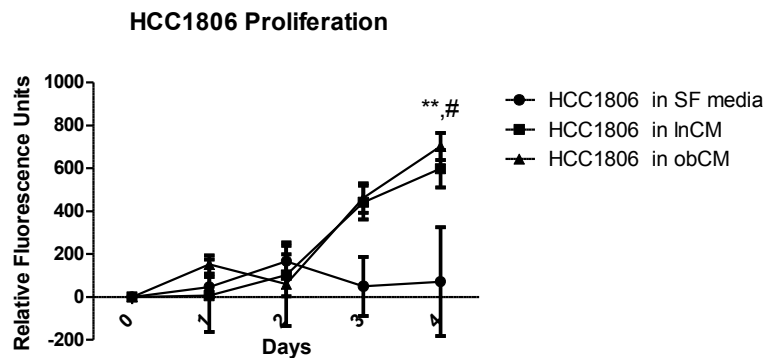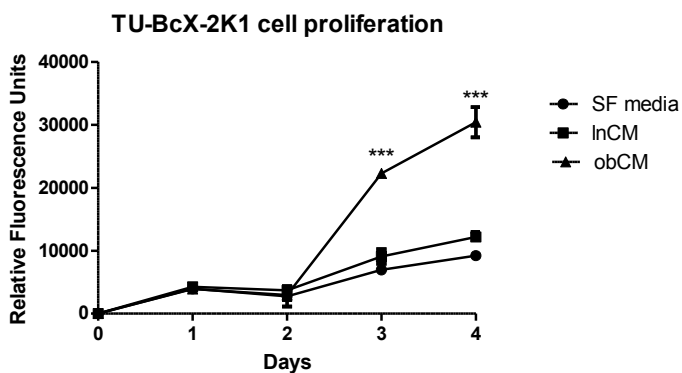

Supplement: Supplementary file 1 — Figure S1. Secreted factors from adipose stem cells promotes proliferation and migration of breast cancer. Conditioned media collected from ASCs after 24 h promotes proliferation of MDA-MB-231, MDA-MB-468, BT20, and HCC1806 TNBC cell lines and TU-BcX-2 K1-derived cells. Values reported are the mean of three independent experiments each performed in triplicate. Bars, ± SEM. *p < 0.05, **p < 0.01, ***p < 0.001. *−obASC vs. control, #−lnASCs vs control (PDF 112 kb) [file 13058_2019_1153_MOESM1_ESM.pdf]

**A**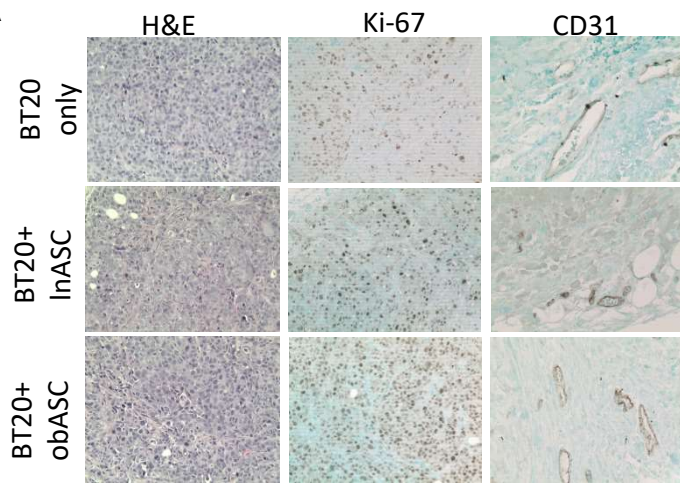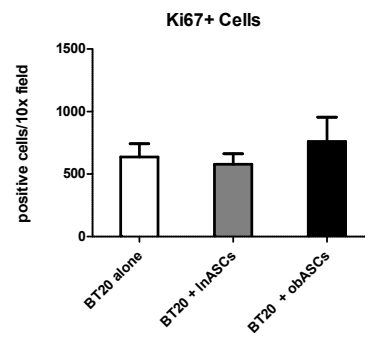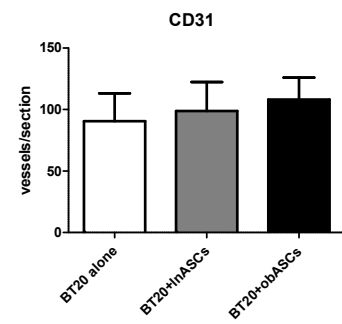**B**

BT20 alone

BT20+InASCs

BT20+obASCs

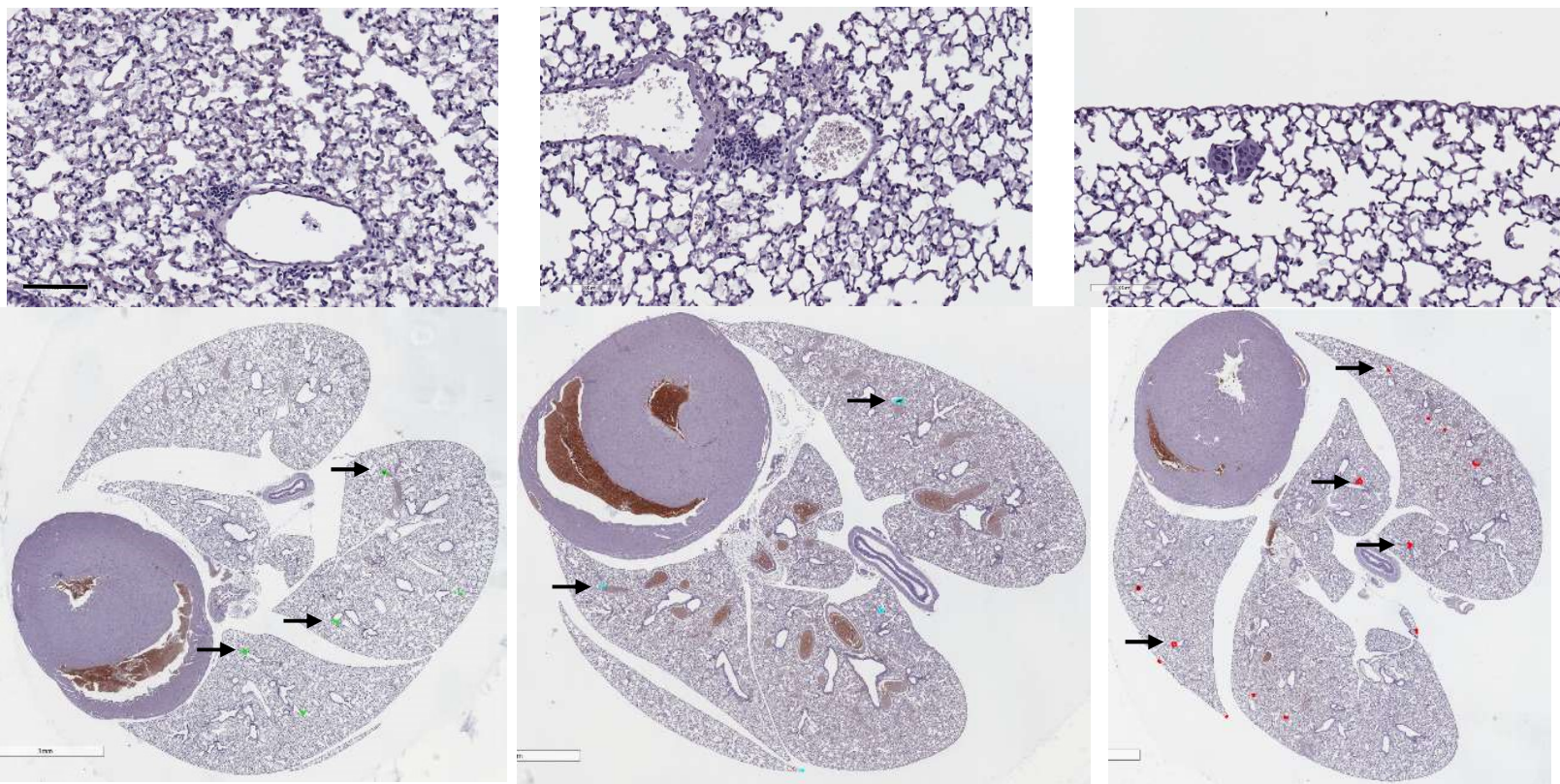**Supplementary Figure 2**

Supplement: Supplementary file 2 — Figure S2. TNBC xenograft growth is unaffected by ASCs. (A) Histologic analysis of tumors at end point revealed that stem cells had no effect on proliferation (ki67+ cells) or angiogenesis (CD31+ blood vessels). All images in panel were acquired at the same magnification. Scale bar represents 100 μm. (B) Representative images of metastases are shown. Image of lung section with annotation metastases are shown. Black arrows point to annotations on lung sections. All images in this panel were acquired at the same magnification. Scale bar represents 100 μm. Values reported are the mean (n = 5 mice per group). Bars, ± SEM. *p < 0.05, **p < 0.01, ***p < 0.001. (PDF 671 kb) [file 13058_2019_1153_MOESM2_ESM.pdf]

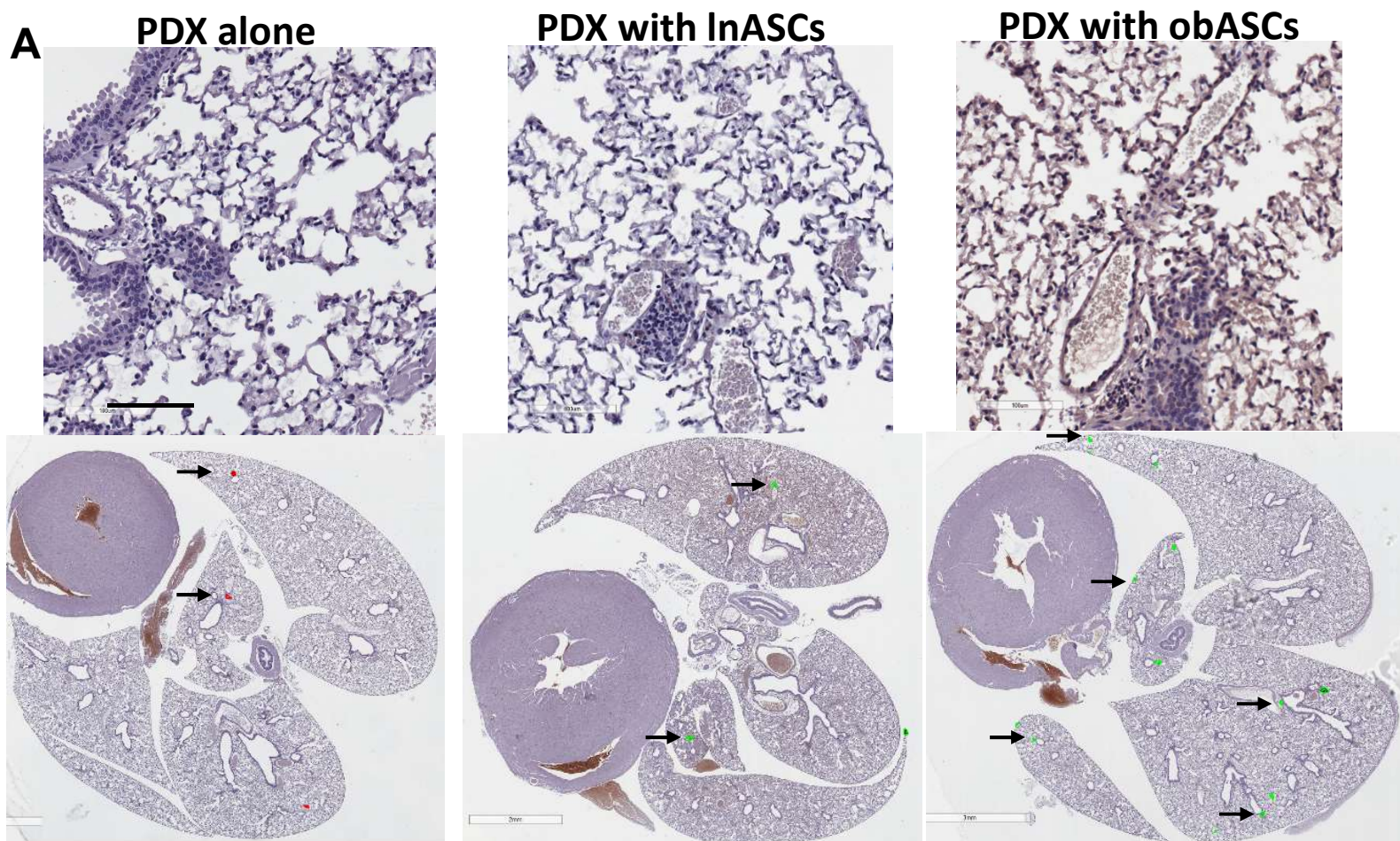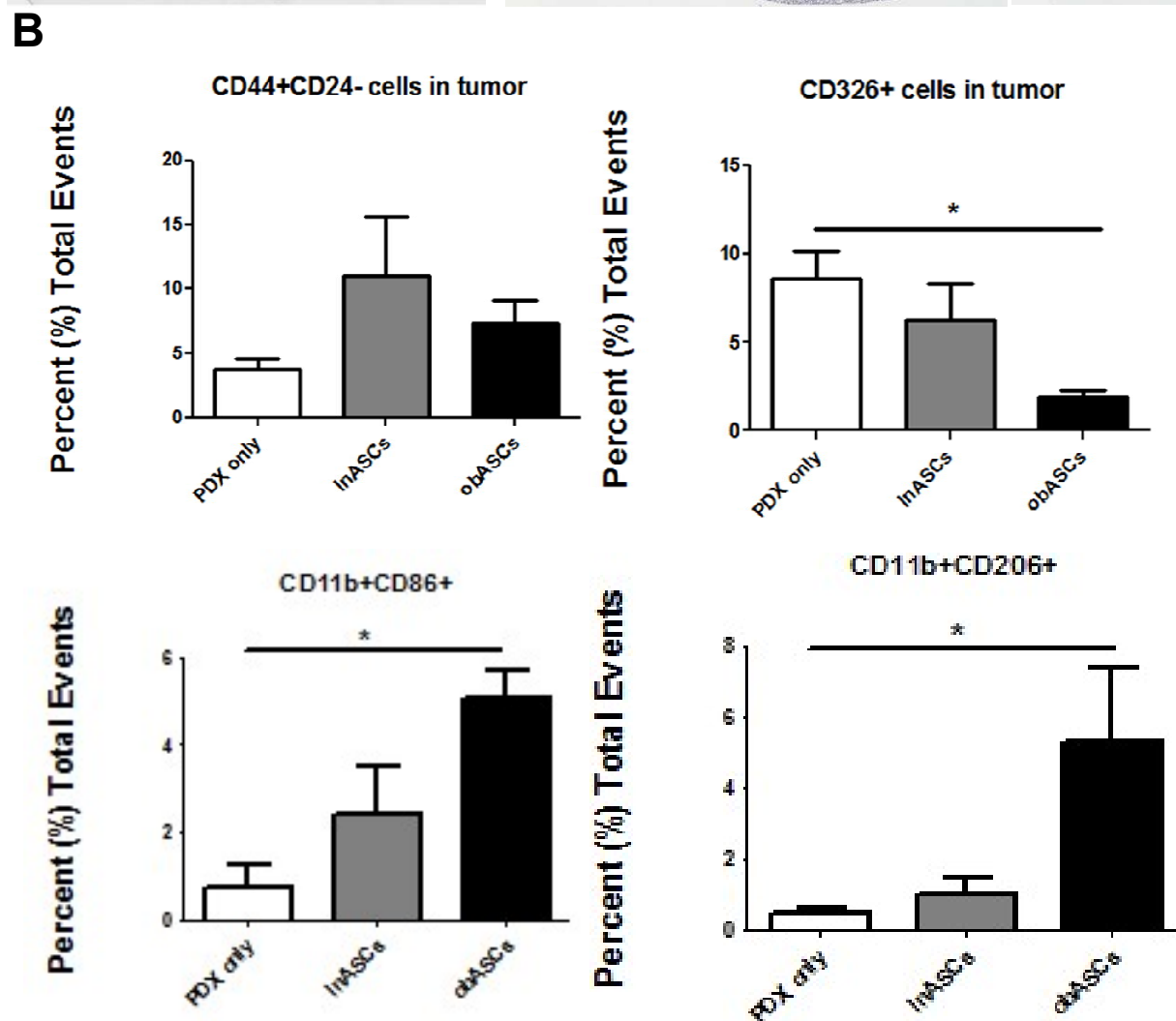

Supplemental Figure 4

Supplement: Supplementary file 4 — Figure S4. Flow cytometric analysis of PDX tumor shows obASCs promote EMT and increase classically activated macrophages and alternatively activated macrophages. (A) Representative images of metastases are shown all and were acquired at the same magnification (scale bar = 100 μm). Image of lung section with annotation metastases are shown. Black arrows point to annotations on lung sections. (B) PDX tumors were digested using 0.01% collagenase to a single-cell suspension, and cells were analyzed using flow cytometry. Tumors had no significant change in cancer stem cell enrichment marker CD44+CD24−, but tumors from the obASC group showed decreased expression of CD326 (epithelial cell adhesion molecule). Analysis of myeloid cells in circulation revealed that obASCs increase the circulating myeloid cells (CD11b+). obASCs significantly increase classically activated macrophages (CD11b+CD86+) and alternatively activated macrophages (CD11b+CD206+) cells. Mean values are represented (n = 5 mice/group) Bars, ± SEM. *p < 0.05. (PDF 1452 kb) [file 13058_2019_1153_MOESM4_ESM.pdf]

**A**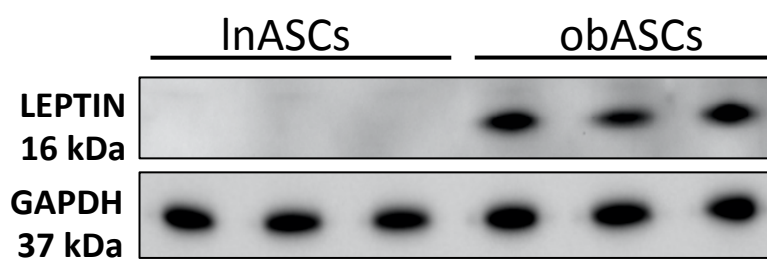**B**

Leptin expression

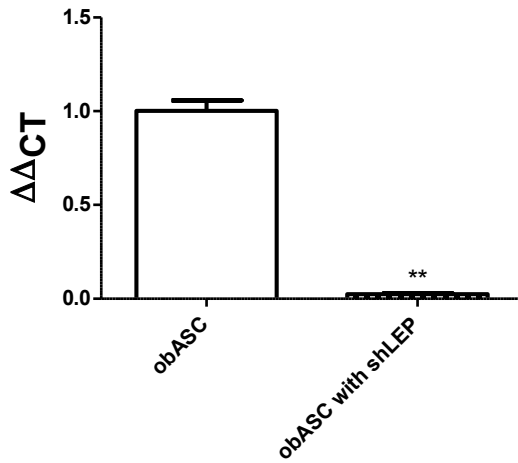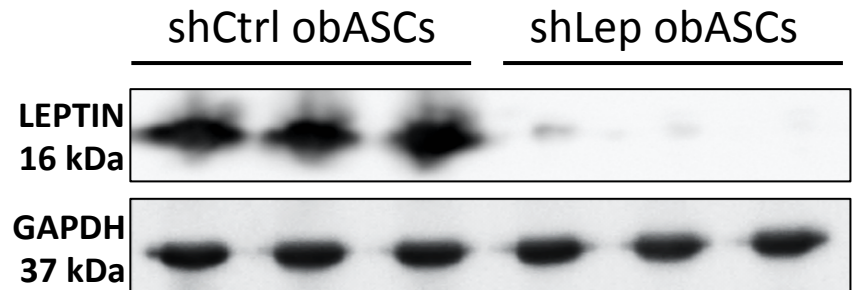**Supplemental Figure 5**

Supplement: Supplementary file 5 — Figure S5. shRNA knockdown of leptin in obASCs. (A) Representative images of metastases are shown and were acquired at identical magnification (scale bar = 100 μm). (B) Leptin expression in control shRNA obASCs versus leptin shRNA obASCs was compared to evaluate knockdown efficiency. Leptin knockdown was confirmed via Western blot. Values reported are the mean of three independent experiments each performed in triplicate. Bars, ± SEM. *p < 0.05, **p < 0.01. (PDF 278 kb) [file 13058_2019_1153_MOESM5_ESM.pdf]

## MCF7

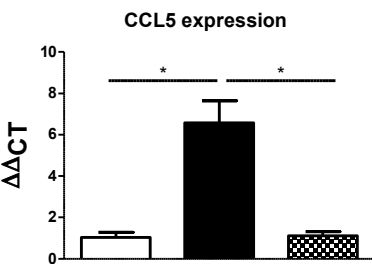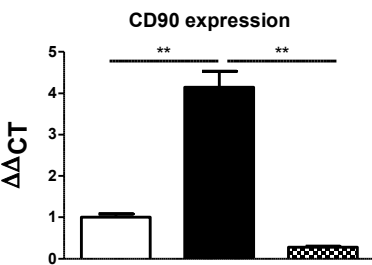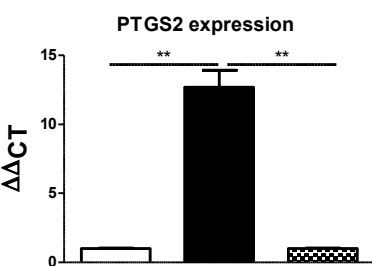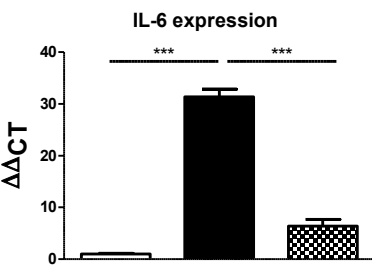

## BT20

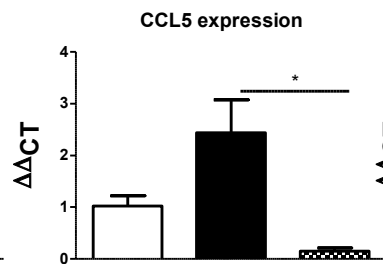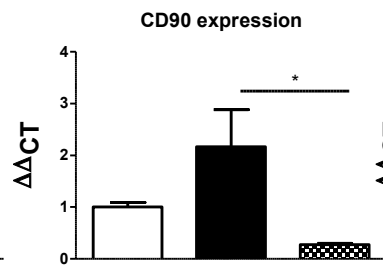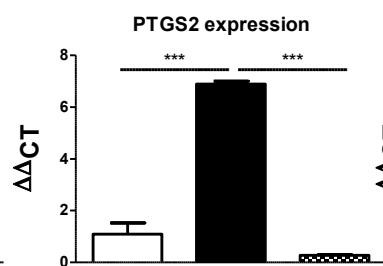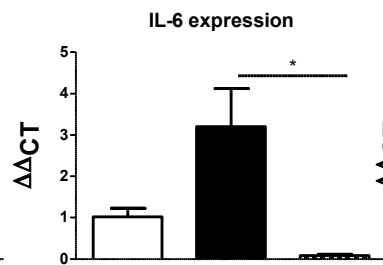

## HCC1806

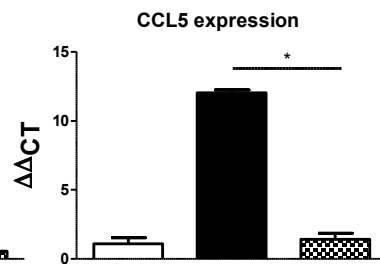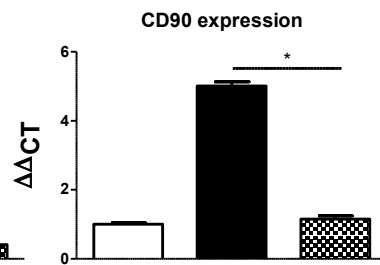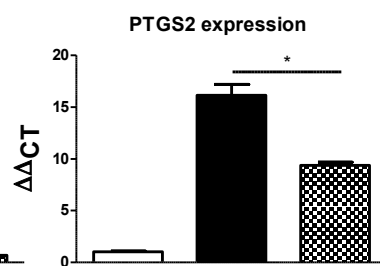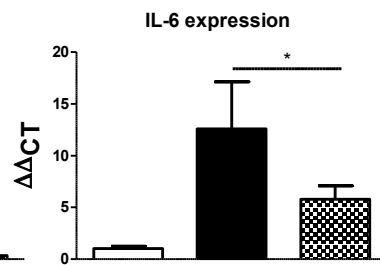

## PDX-derived cells

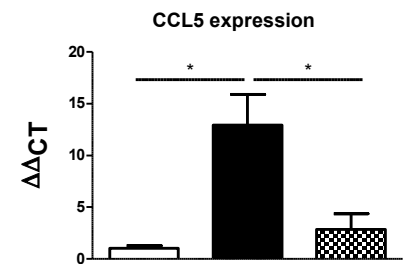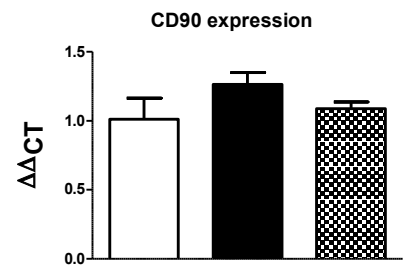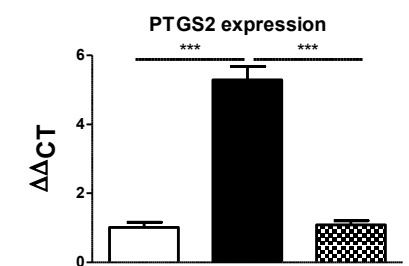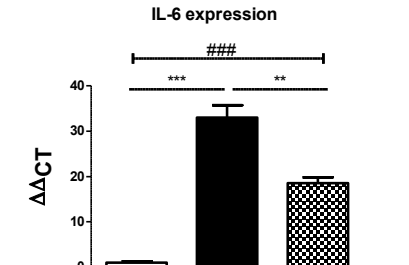

Supplement: Supplementary file 6 — Figure S6. shRNA knockdown of leptin in obASCs decreases pro-metastatic gene expression in breast cancer cells. RT-qPCR of breast cancer cells after 3 days of transwell co-culture with control shRNA obASCs or leptin shRNA obASCs shows an increase in expression of CCL5, CD90, PTGS2, and IL-6 after co-culture with obASCs that is abrogated by leptin shRNA in the obASCs. Values reported are the mean of three independent experiments each performed in triplicate. Bars, ± SEM. *p < 0.05, **p < 0.01, ***p < 0.001. (PDF 182 kb) [file 13058_2019_1153_MOESM6_ESM.pdf]

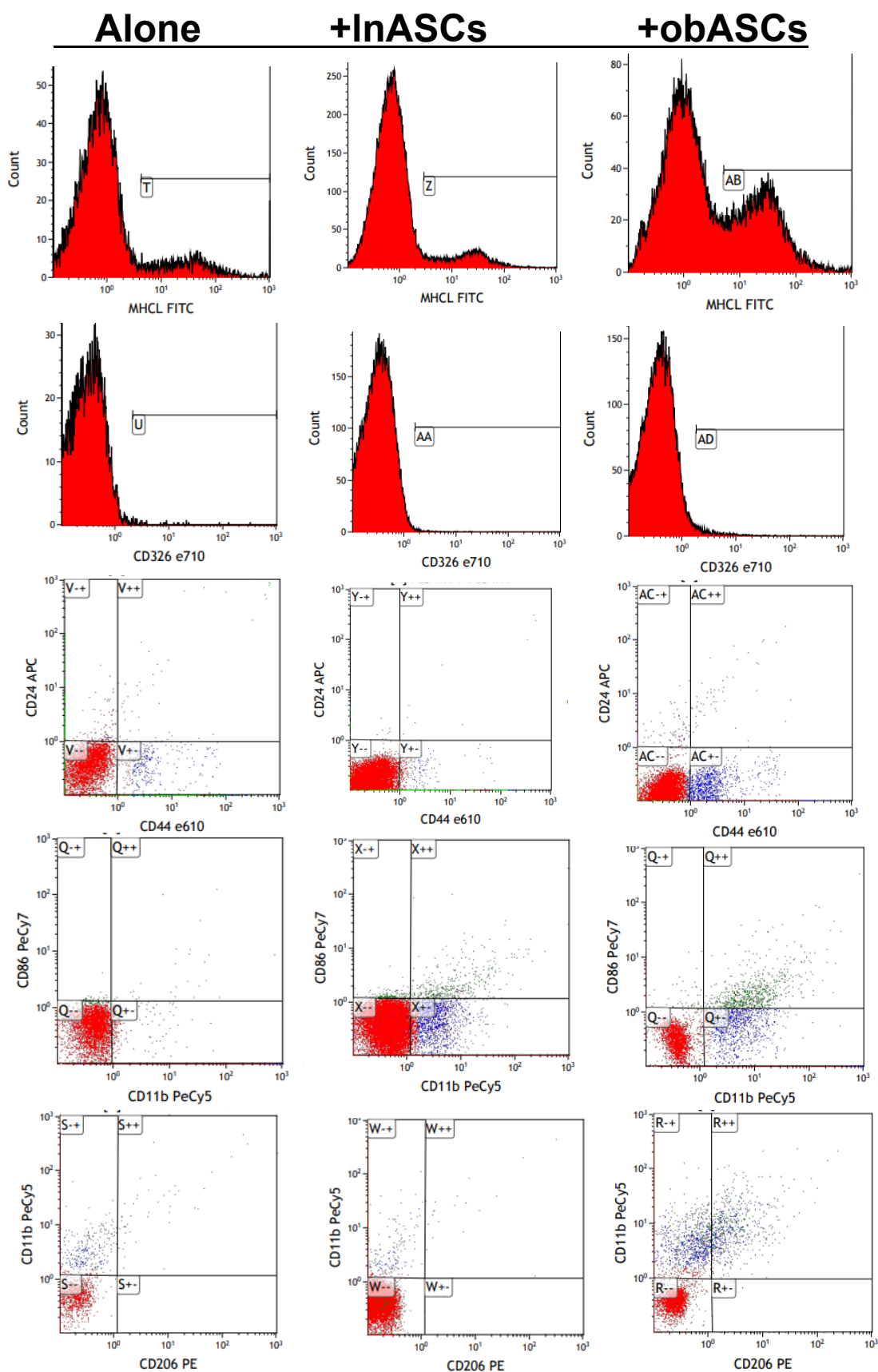

Supplemental Figure 7

Supplement: Supplementary file 7 — Figure S7. Flow cytometry gating strategy. Representative dot plots and histograms generated from Beckman Coulter Galios Flow Cytometer are shown here to represent the gating strategies for flow cytometry analysis included in this manuscript. (PDF 361 kb) [file 13058_2019_1153_MOESM7_ESM.pdf]
