# Supplementary material for: Leptin produced by obesity-altered adipose stem cells promotes metastasis but not tumorigenesis of triple-negative breast cancer in orthotopic xenograft and patient-derived xenograft models
Source: Breast Cancer Res. 2019 May 22;21:67. doi: 10.1186/s13058-019-1153-9 (PMC6530039; doi:10.1186/s13058-019-1153-9)
Supplement: Supplementary file 3 — Figure S3. Patient-derived xenograft. (A) Primary human TNBC was surgically removed and implanted with Matrigel into SCID/Beige female mice (n = 5 per group). SCID/Beige mice lack B cells, T cells, and functional natural killer cells. At passage seven, tumors pieces were implanted with lnASCs, obASCs, or PDX alone. (B) Analysis of immunostaining of tumors at end point revealed that ASCs had no effect on proliferation (ki67+ cells) or angiogenesis (CD31+ blood vessels). All images in panel acquired at the same magnification. Scale bar represents 200 μm. (C) Histologic analysis CD44+ is shown. Scale bar in upper row represents 60 μm and 20 μm in the lower panel. (D) Histologic analysis CD44+, CD36+, and CPT1 is shown. All images were acquired at the same magnification. Scale bar represents 10 μm. Values reported are the mean (n = 5 mice/group). Bars, ± SEM. *p < 0.05, **p < 0.01, ***p < 0.001. (PDF 10977 kb) [file 13058_2019_1153_MOESM3_ESM.pdf]

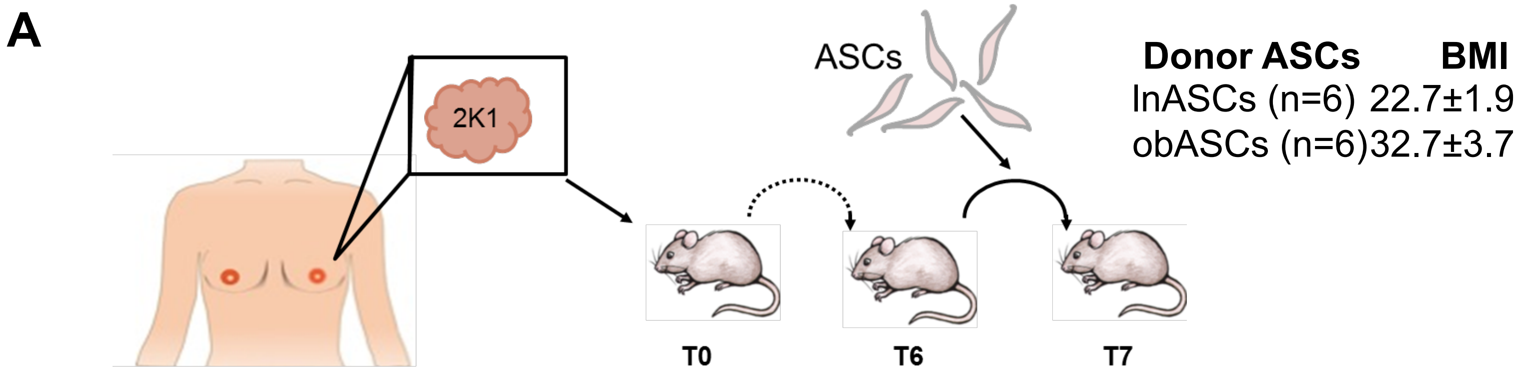

PDX no.	Age	Race	PAM50 molecular subtype	Histologic type	Ki67	Lymph node involvement	Distant metastases
2K1	59	AA	TNBC	Invasive ductal carcinoma	>99%	N	N

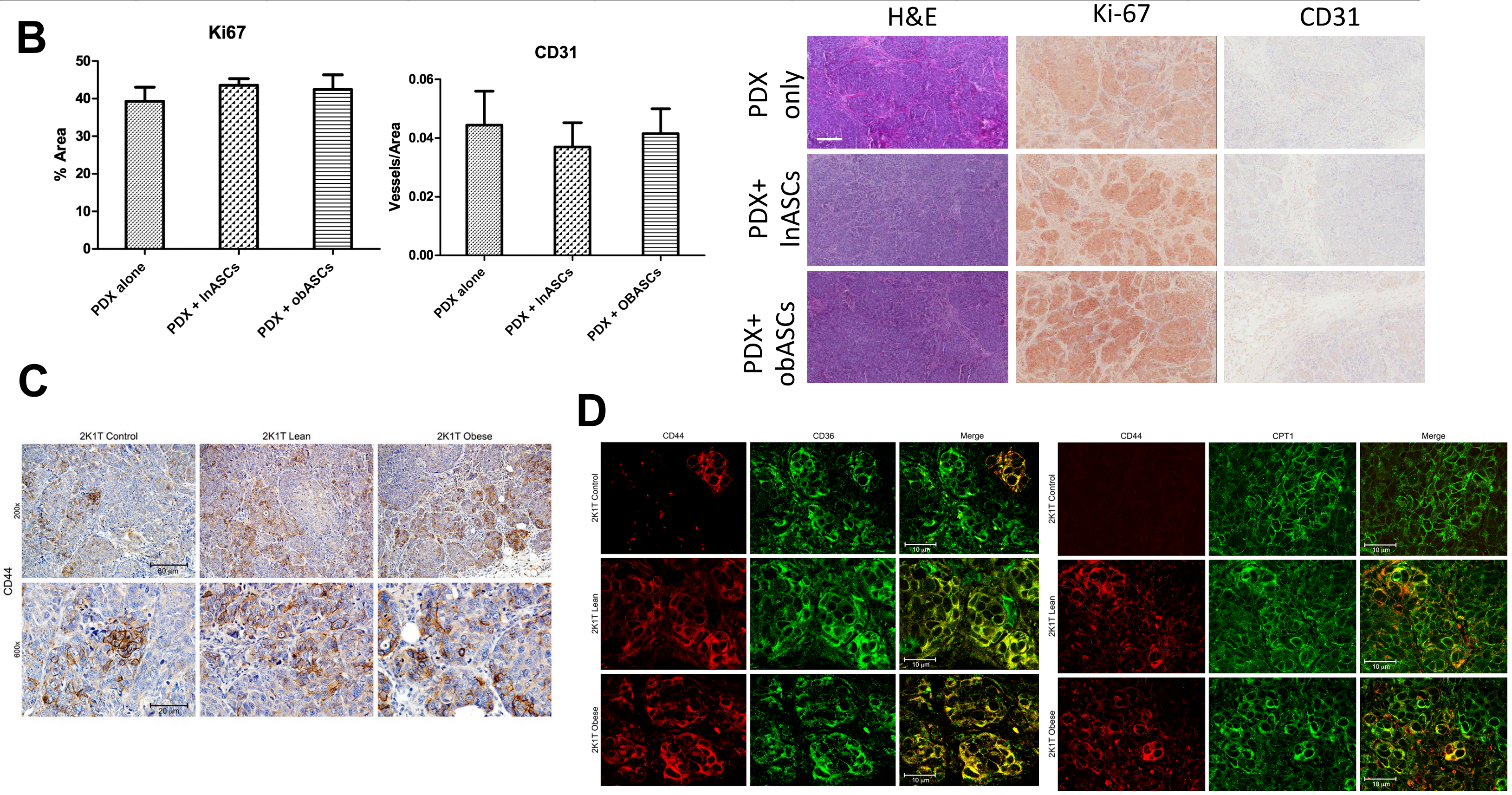

Supplemental Figure 3
